# Single-Atom Cobalt-Based Electrochemical Biomimetic Uric Acid Sensor with Wide Linear Range and Ultralow Detection Limit

**DOI:** 10.1007/s40820-020-00536-9

**Published:** 2020-10-27

**Authors:** Fang Xin Hu, Tao Hu, Shihong Chen, Dongping Wang, Qianghai Rao, Yuhang Liu, Fangyin Dai, Chunxian Guo, Hong Bin Yang, Chang Ming Li

**Affiliations:** 1 Institute of Materials Science and Devices, Suzhou University of Science and Technology, Suzhou, 215009 People’s Republic of China; 2 Institute for Advanced Cross-field Science and College of Life Science, Qingdao University, Qingdao, 200671 People’s Republic of China; 3 Institute for Clean Energy and Advanced Materials, School of Materials and Energy, Southwest University, Chongqing, 400715 People’s Republic of China; 4 School of Chemistry and Chemical Engineering, Southwest University, Chongqing, 400715 People’s Republic of China; 5 Suzhou Institute of Biomedical Engineering and Technology, Chinese Academy of Sciences, Suzhou, 215163 People’s Republic of China; 6 State Key Laboratory of Silkworm Genome Biology, College of Biotechnology, Southwest University, Chongqing, 400715 People’s Republic of China

**Keywords:** Single-atom cobalt, Nanozyme, Biocatalysis, Uric acid, Molecular interaction

## Abstract

Uric acid (UA) detection is essential in diagnosis of arthritis, preeclampsia, renal disorder, and cardiovascular diseases, but it is very challenging to realize the required wide detection range and low detection limit. We present here a single-atom catalyst consisting of Co^(II)^ atoms coordinated by an average of 3.4 N atoms on an N-doped graphene matrix (A–Co–NG) to build an electrochemical biomimetic sensor for UA detection. The A–Co–NG sensor achieves a wide detection range over 0.4–41,950 μM and an extremely low detection limit of 33.3 ± 0.024 nM, which are much better than previously reported sensors based on various nanostructured materials. Besides, the A–Co–NG sensor also demonstrates its accurate serum diagnosis for UA for its practical application. Combination of experimental and theoretical calculation discovers that the catalytic process of the A–Co–NG toward UA starts from the oxidation of Co species to form a Co^3+^–OH–UA*, followed by the generation of Co^3+^–OH + ^*^UA_H, eventually leading to N–H bond dissociation for the formation of oxidized UA molecule and reduction of oxidized Co^3+^ to Co^2+^ for the regenerated A–Co–NG. This work provides a promising material to realize UA detection with wide detection range and low detection limit to meet the practical diagnosis requirements, and the proposed sensing mechanism sheds light on fundamental insights for guiding exploration of other biosensing processes.

**Electronic supplementary material:**

The online version of this article (10.1007/s40820-020-00536-9) contains supplementary material, which is available to authorized users.

## Highlights

- A single-atom catalyst of A–Co–NG is explored for electrochemical uric acid (UA) detection for the first time and realize practical UA monitoring in serum samples.
- The A–Co–NG sensor demonstrates high performance for UA detection with a wide detection range from 0.4 to 41950 μM and an extremely low detection limit of 33.3 nM.
- Combination of experimental and theoretical calculation discovers mechanism for the UA oxidation on the single-atom catalyst.

**Electronic supplementary material:**

The online version of this article (10.1007/s40820-020-00536-9) contains supplementary material, which is available to authorized users.

## Graphical abstract

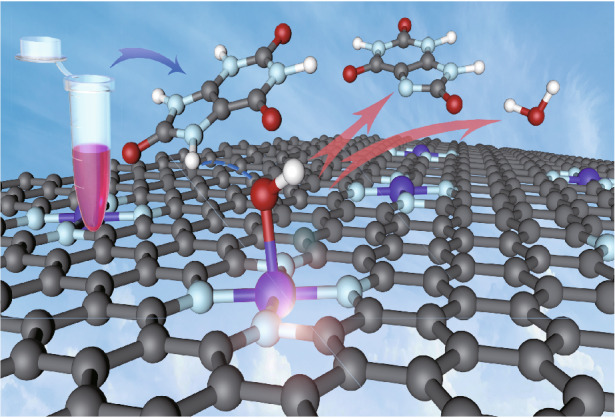

## Introduction

Uric acid (UA) is the metabolization of purine alkaloids [1, 2], and is recognized as an important biomarker for diseases such as arthritis, preeclampsia, renal disorder, and cardiovascular diseases [3–6]. The realization of UA detection is essential in diagnosing the diseases discussed above. Various methods have been developed for diagnosis of UA, which includes colorimetric enzymatic assays [7], liquid chromatography [8], capillary electrophoresis methodologies [9], surface enhanced Raman scattering [10], and electrochemical method [11]. Among these methods, electrochemical detection offers simplicity in operation, fast response, high sensitivity, low cost, and potential in miniaturization. Currently developed electrochemical UA sensors are always based on enzymes such as uricase that have high cost, poor stability and harsh storage conditions [12, 13]. The enzyme sensors involve complex steps of UA decomposition to form allantoin and H_2_O_2_, which are subsequently catalyzed to realize detection [14], restricting the clinical applications. More critically, UA concentrations have a very wide range in human bodies [15]. For example, the normal concentration range of UA in human blood is 15 to 80 mg L^−1^. While for people suffers from the urate nephropathy and gout infection, the UA in human blood is as low as 6 mg L^−1^. Moreover, UA levels in kidney stones can vary widely from day to day. Thus, the realization of enzyme-free sensing of UA with wide detection range and low detection limit is critical in clinical applications for diagnosis of the related diseases.

Recent efforts have been spent in exploring nanostructured materials to replace enzyme in electrochemical detection of UA, and some examples are Prussian blue (PB)/N-doped CNTs [13], polyacrylamide-coated CNT [16], Au nanocrystals anchored on graphene oxide (GO) [17], mesoporous Co_3_O_4_ [18], ZnO/Ag_2_O/Co_3_O_4_ [19], and g-Ce_2_S_3_-CNT [20]. Although they could overcome drawbacks of enzyme-based sensors, the nanostructured materials-based ones still suffer from relatively narrow detection range and poor detection limit. The relatively poor sensing performance should be attributed to their low density of exposed active sites. Besides, nanostructured materials show an inhomogenous elemental composition and facet structure, resulting in different and complicated catalytic mechanisms. Single-atom catalysts (SACs) that are defined as atomically dispersive activity sites have demonstrated promising applications owing to their advantages of homogeneous active sites, high metallic atom utilization and fast catalytic kinetic [21–23], which could bridge the gap between natural enzyme and nanozyme and understanding of the catalytic mechanism. SACs have been applied in various catalytic reactions since the report of Pt atoms on FeO_*x*_ with high CO oxidation activity [24]. In particular, as a kind of SACs, nitrogen-doped carbon supported SACs (e.g., Metal–Nitrogen–Carbon shorten as M–N–C) have attracted great attention very recently because of their large specific surface area, high active site density, and good electrical conductivity [25, 26]. By arranging N and metal atoms, the M–N–C SACs possess similar M–Nx active sites as natural metalloenzymes, enabling enzyme-like behaviors [27]. For example, a SAC of carbon nanoframe-confined FeN_5_ single active centers behaves as oxidase-like activity toward 3,3′,5,5′-tetramethylbenzidine [28]. Considering the enzyme-like activity together with high active site density and good electrical conductivity, it is expected that M–N–C SACs could be used as functional materials in electrochemical detection of UA to achieve long detection range and low detection limit. Among the transition metal (Co, Mn, Fe, Ni, and Cu) SACs, Co-SAC has been reported to behave the optimal d-band centers, which can function as a highly active and selective catalyst [25]. Nevertheless, such a possibility has not been explored yet.

In this work, we present the fabrication of a M–N–C SAC comprising high-density and isolated cobalt atoms anchored on an N-doped graphene matrix (shorten as A–Co–NG), which is the first report of SACs in electrochemical sensing of UA. Material characterizations, experiments and theoretical investigations are carried out to elucidate the structure, properties, enzyme-like electrochemical activity of A–Co–NG and catalytic mechanisms as well as substrate affinity and corresponding reaction energies. Results showed the single Co atom nanozyme exhibits high intrinsic enzyme-like activity, fast response and good selectivity toward UA oxidation compared with that of recently reported works due to its abundant and efficient activity sites. Eventually, the A–Co–NG-based electrochemical sensor shows a long detection range and low detection limit toward UA. This work demonstrates a great approach for rationally designing high-efficient biomimetic nanozymes while offering scientific insights for understanding of intrinsic physiochemical mechanism of single-atom nanozymes.

## Experimental Section

### Materials

Graphene oxide was synthesized from graphite flakes using the improved Hummers method [29]. Sodium hydroxide (NaOH), cobalt chloride (CoCl_2_·6H_2_O), cobalt nitrate (Co(NO_3_)_2_·6H_2_O), cobalt acetate ((CH_3_COO)_2_Co), uric acid (UA), melamine, glutamic acid, ascorbic acid (AA), dopamine (DA), sodium sulfate (Na_2_SO_4_), potassium chloride (KCl), glucose (Glu) sulfuric acid (H_2_SO_4_), sodium nitrite (NaNO_2_), potassium hydroxide (KOH), potassium ferricyanide (K_3_[Fe(CN)_6_]), potassium ferrocyanide (K_4_[Fe(CN)_6_]) and Nafion were purchased from Sigma-Aldrich. Nitric oxide (NO) was prepared through the reaction between H_2_SO_4_ and NaNO_2_ and purified with different concentrations of KOH. Buffer solution was prepared using Mettler-Toledo pH meter. All of the other chemical reagents were purchased from Sigma-Aldrich and used directly without further purification. Milli-Q water (resistivity over 18 MΩ cm) from a Millipore-Q water purification system was used in all experiments.

### Apparatus

The crystal structure, morphology and chemical composition of the samples were analyzed by scanning electron microscopy (SEM, Zeiss Merlin, Germany), transmission electron microscopy (TEM, FEI F20, USA) and energy dispersive X-ray spectroscopy (EDS, JEOL JED-2300 Analysis Station, Japan). X-ray photoelectron spectroscopy (XPS) measurements were carried out on an ESCALAB 250Xi photoelectron spectrometer (Thermo Fisher Scientific, USA) at 2.4 × 10^10^ mbar using a monochromatic Al Kα X-ray beam (1486.60 eV). All measured binding energies were referenced to the C 1s peak (284.60 eV) arising from the adventitious hydrocarbons. N_2_ adsorption–desorption isotherms were conducted on an 3H-2000PS1 accelerated surface area and porosimetry system (Bei Shi De, China) at 77 K using Barrett–Emmett–Teller (BET) calculations for the surface area. The pore size distribution plot was determined with the desorption branch of the isotherm on the Barrett–Joyner–Halenda (BJH) model. X-ray diffraction (XRD) was conducted at Bruker D8 advance (Germany). Electrochemical measurements were performed in 0.1 M NaOH (pH = 13) on a CHI 760e electrochemical workstation (CH Instruments, Chenhua Corp., China). Three-electrode setup was employed with Pt plate (1.0 × 1.0 cm^2^) and saturated calomel electrode (SCE) as the counter and reference electrode, respectively. And a working electrode was prepared by using different materials modified electrode. The metal contents of the catalysts were measured by ICP-MS, which were carried out by a Thermo Scientific iCAP6300 (Thermo Fisher Scientific, USA). X-ray absorption spectra were collected at Shanghai Synchrotron Radiation Facility (SSRF) on beamline BL14W1. All the data were collected in the transmission mode at ambient temperature. Data analysis was performed with Artemis and IFEFFIT software [30, 31].

### Synthesis of A–Co–NG

Initially, 250 mg GO was added into 100 mL deionized water under continue sonicating to prepare an aqueous suspension of GO. Then, (CH_3_COO)_2_Co was added in GO suspension with a mole ratio as GO: Co = 125: 1, the mixture was sonicated for another 2 h, and subsequently mixed with 500 mg melamine through ball milling, followed by freeze-dried for at least 24 h. The dried sample was placed in the center of a standard 1-inch quartz tube furnace. After pumping and purging the system with argon three times, the temperature was ramped at 20 °C up to 800 °C for 2 h with a heating rate of 3 °C min^−1^ under the feeding of argon at ambient pressure. The final product A–Co–NG with a blackish color was obtained after the furnace and permitted to cool to room temperature under argon protection. Particle Co metal modified NG (P–Co–NG) was synthesized with the same procedure under a mole ratio of GO: Co as 50: 1.

### Synthesis of Co_3_O_4_/GO, Co_3_O_4_ and NG

Co_3_O_4_/GO nanocomposites were synthesized by mixing 20 mL 9 mg mL^−1^ GO with 3.6 mg (CH_3_COO)_2_Co (with a molar ratio of GO: Co as 50:1) under intense stirring for 30 min, then the mixture was added in 20 mL 0.1 M NaOH solution and stirred for another 30 min. The obtained solution was transferred into 100 mL autoclave with a Teflon liner at 180 °C, and kept for 24 h. The obtained product was filtered, and then washed with H_2_O and ethanol for several times, then dried naturally in air. Co_3_O_4_ nanomaterial was obtained with the same procedure without adding GO solution [32]. Nitrogen-doped graphene (NG) was obtained by annealing melamine with glutamic acid under N_2_ protection.

### Fabrication of the Modified Electrode

To prepare the UA biosensor, a disk glass carbon electrode (GCE) with a diameter as 3 mm was applied as the substrate, which was sequentially polished by 0.3 and 0.05 µm alumina, followed by successive ultrasonication with distilled water and ethanol for 2 min until obtaining a mirror like surface. Then, with aid of ultrasonic, 5.0 mg mL^−1^ A–Co–NG suspension was prepared applying ethanol and deionized water mixture (1:1) as a dispersing agent. Subsequently, 5 µL A–Co–NG suspensions (25 µg) were dropped on clean GCE surface and dried in room temperature to obtain A–Co–NG/GCE. The thickness of the film was measured using SEM, showing a value of 14 ± 0.04 µM. The thickness of the A–Co–NG film is quite uniform as confirmed by measuring different locations of the prepared electrode. The final electrode was applied to detect UA. For comparison, Co_3_O_4_/GO/GCE, Co_3_O_4_/GCE, NG/GCE, and P–Co–NG/GCE were also prepared with same procedure for preparation of A–Co–NG/GCE.

### Real Sample Detection

For real sample analysis, drug-free human serum samples were collected from healthy volunteers from Xinqiao Hospital (Chongqing, China). All experiments were conducted in good compliance with the relevant laws and institutional guidelines. The serum samples were treated by centrifugation and filtration to remove large-size proteins, and then diluted 5 times with 0.01 M PBS. Then, standard addition method, commonly used to eliminate background effects on various sourced samples for measurement accuracy, was applied to conduct real sample detection. The method is performed by reading the electrochemical current responses of the serum samples, and then by measuring the current responses of the unknown sample with an amount of known standard added. In diagnosis, 250 µL diluted serum sample was added into 5 mL 0.1 M NaOH followed by adding 10 µL of 5 mM UA into the same serum sample to prepare a spiked one. The amperometric *I−t* measurements were performed before and after the addition of known concentrated UA with A–Co–NG/GCE, respectively. The recovery was calculated according the following equation:

$${\text{Recovery}} = \left( {C_{2} - C_{1} } \right)/C_{3} \times 100\%$$

*C*_1_ and *C*_2_ are concentrations of serum and spiked samples, respectively, which are calculated from the calibration curve. *C*_3_ stands for concentration of standard addition of UA.

### Models and Computational Details for DFT

All the calculations in this work are carried within the framework of density functional theory (DFT) using the Vienna Ab initio Simulation Package (VASP) [33]. The exchange correlation energy was modeled by using the Perdew–Burke–Ernzerhof (PBE) functional within the generalized gradient approximation (GGA) [34]. Projector augmented wave (PAW) pseudopotentials [35] were used to describe ionic cores, while electron–ion interactions were described by ultrasoft pseudopotentials. A 15 Å vacuum was inserted in the *z* direction to prevent image interactions. The cutoff energy was 500 eV. To exclude the image effect in periodic models, a 6 × 6 supercell of graphene with in-plane lattice parameters > 10 Å was used to construct models of Co-N_4_-doped and N-doped graphene. The k-point sampling employs a 3 × 3 × 1 mesh within the Monkhorst–Pack scheme [36]. For the calculation of reaction intermediates, the van der Waals interaction is considered by the long-range interaction dispersive correction (DFT-D) method [37].

## Results and Discussion

### Structure Characterization of A–Co–NG

**Fig. 1:**
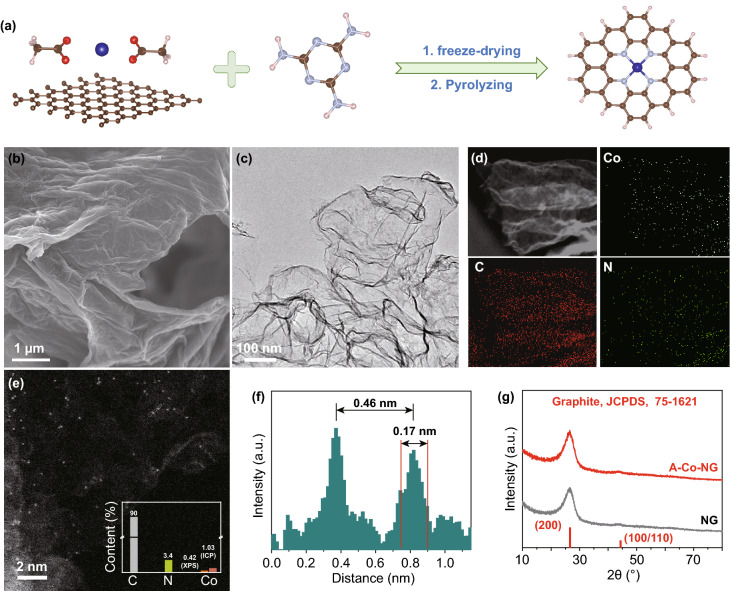
**a** Schematic illustration of the synthetic procedure of the A–Co–NG nanozyme. Structural characterization of A–Co–NG: **b** SEM image; **c** Bright-field TEM image; **d** EDX mapping images; **e** HAADF-STEM image, inset is content of atoms; **f** Statistic distance between adjacent Co bright spots; **g** XRD diffraction patterns of A–Co–NG and NG

The A–Co–NG catalyst was prepared by absorbing (CH_3_COO)_2_Co on GO and then mixing the composite with melamine through ball milling. Finally, the mixture was pyrolyzed in argon, as showed in Fig. 1a. SEM and TEM were applied to character its morphology and structure. The as-prepared A–Co–NG nanomaterial behaves a similar morphology feature to graphene with sheet-like structures with smooth surface (Fig. 1b, c). The referenced catalysts like P–Co–NG, NG, Co_3_O_4_, and Co_3_O_4_/GO composites were also characterized by SEM and TEM as shown in Figs. S1, S2. The homogeneous distributions of Co and N atoms are highlighted by the elemental mapping measurement (Fig. 1d), which indicates uniformly distribution of Co and N atoms throughout in carbon matrices. The HAADF-STEM image (Fig. 1e) exhibits isolated high-density bright spots distribute across the entire carbon framework in A–Co–NG, which corresponding to single Co atom has larger atomic mass than C. The content of Co atom in A–Co–NG is 1.03% determined by ICP (inset of Fig. 1e). The sizes of the bright spots are ~ 0.17 nm, and the statistic distance between adjacent bright spots (~ 0.46 nm) is larger, as shown in Fig. 1f. The atomic dispersion of Co atoms on graphene support was further confirmed by the XRD pattern. As shown in Fig. 1g, only (200) and (100/110) carbon diffraction peaks at 26.2° and 44.0° are observed, revealing no Co-derived particles or characteristic crystal peaks of Co are formed. Figure S3 displayed XRD patterns of P–Co–NG, NG, Co_3_O_4_, and Co_3_O_4_/GO composites, from which typical crystal peaks of Co could be observed. BET investigation indicates A–Co–NG obtains a large surface area up to 816.108 m^2^ g^−1^ and numerous mesopores with a mean pore size of 3.931 nm (Fig. S4).

**Fig. 2:**
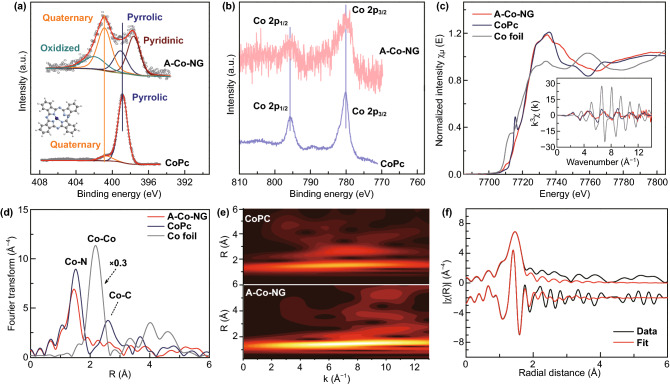
Characterization of the single-atom catalysts. XPS spectra of **a** N 1s and **b** Co 2p for A–Co–NG and CoPc, respectively; **c** K-edge XANES spectra of A–Co–NG, inset is the *k*3-weighted k-space spectra; **d** Fourier transformed (phase uncorrected) Co K-edge EXAFS spectra; **e** wavelet transform of the *k3*-weighted EXAFS spectrum of the A–Co–NG and CoPc; **f** First-shell fitting of the Fourier transformation of the EXAFS spectrum of A–Co–NG (the EXAFS spectrum was fitted using the FEFF 8.2 code)

The chemical composition and elemental states of Co atoms in samples were firstly investigated by XPS as shown in Figs. 2a, b and S5. The binding energy of Co 2p_3/2_ in A–Co–NG is at 789.6 eV, which slightly shift ~ 0.25 eV relative to the cobalt phthalocyanine (CoPc) (II), indicating similar valence states of Co for tow samples. From the high-resolution XPS N 1s spectrum of CoPc (II), the major peak at 398.85 eV was assigned to pyrrolic, which linked with Co atom. A–Co–NG was deconvoluted into pyridinic (~ 398.05 eV), pyrrolic (~ 399.5 eV), quaternary (~ 401.15 eV), and oxidized (~ 402.3 eV) N species [38]. It could be deduced that pyridinic N mainly connected with Co atom in A–Co–NG from Fig. 2a. The chemical states of Co atoms in A–Co–NG was further investigated by the X-ray absorption spectra (XAS) (Fig. 2c, d). Figure 2c shows the K-edge X-ray absorption near edge spectra (XANES) of A–Co–NG and reference samples. The rising edge of Co absorption for A–Co–NG is 7722.3 eV which is exactly same with that of CoPc, indicating +2 of oxidation state of Co atoms in the A–Co–NG. As shown in Fig. 2d, the coordination environment of Co atoms in the A–Co–NG was further analyzed by Fourier transform of extended X-ray absorption fine structure (FT-EXAFS), which shows only one strong shell (1.46 Å), that is 0.06 Å shorter than the Co–N (1.52 Å) bond in the CoPc (II) sample. Moreover, the features of Co–Co bond (~ 2.16 Å) for Co-foil and Co–C bond (~ 2.60 Å) for CoPc (II) are undetectable in the A–Co–NG, confirming atomic dispersed and N atoms coordinated of Co atoms on graphene. The kind of backscattering atoms for the formation of peak at 1.46 Å of A–Co–NG was distinguished by analysis of the wavelet transform (WT) of the k3-weighted EXAFS spectrum. As shown in Fig. 2e, the A–Co–NG and CoPc (II) have the maximums intensity at the same k value (6.5 Å^−1^), indicating the peak of first shell for A–Co–NG origin from same backscattering atoms as that of CoPc (II), that is N atoms. Moreover, the difference of bond length between two samples implies the N species with the Co atom in A–Co–NG is different with pyrrolic N in CoPc (II), which is in agree with the conclusion from differential of XPS N 1s between two samples. The FT-EXAFS of A–Co–NG and CoPc (II) was fitted by the Co-N path (Figs. 2f, S6 and Table S1), the coordination number is about 3.4. Based on the structural characterization and chemical state investigation, the Co atoms in A–Co–NG are atomic dispersed on graphene, in +2 valence state, and coordinated by about 3.4 N atoms, on average.

### Electrocatalytic Behaviors of A–Co–NG toward UA Oxidation

**Fig. 3:**
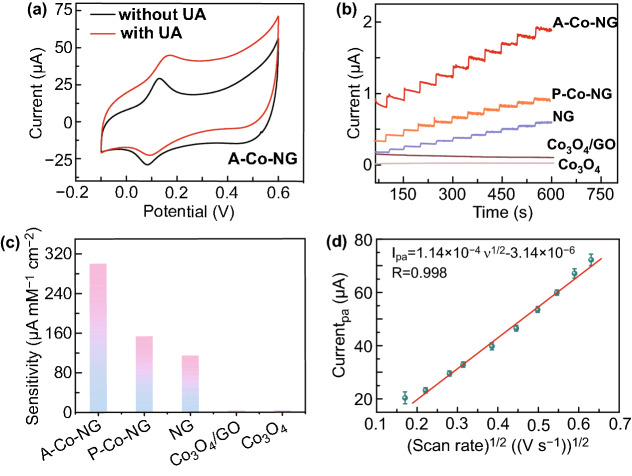
**a** CV curves of the A–Co–NG nanozyme recorded in a 0.1 M NaOH (pH = 13) solution without and with 400 µM UA; **b** Amperometric *I*-*t* response of various catalysts upon continuous injection of 5 µM UA at an applied potential of 0.3 V versus SCE in 0.1 M NaOH (pH = 13); **c** Histogram of sensitivity for UA detection of A–Co–NG, P–Co–NG, NG, Co_3_O_4_/GO, and Co_3_O_4_; **d** Anodic peak currents of the cyclic voltammograms versus the square roots of a various scan rates from 0.03 to 0.4 V s^−1^

The oxidase-like activities of A–Co–NG were determined through electrochemical assays toward UA catalytic reaction. Cyclic voltammetry (CV) curve (Fig. 3a black curve) shows a pair of defined redox peaks in 0.1 M NaOH solution (pH = 13) for A–Co–NG/GCE with oxidation and reduction peak potentials of 1.143 and 1.095 V versus RHE, respectively, which are in good agreement with the standard redox reaction potential of Co(II)/Co(III). After adding 400 μM UA into the 0.1 M NaOH solution (pH = 13), the oxidation current significantly increased, attributing to the oxidation of UA (Fig. 3a red curve). In addition, the response currents of A–Co–NG increases with the increase in UA concentration in a range of 0 to 800 μM, as shown in Fig. S7, indicating an excellent performance of A–Co–NG nanozyme. Furthermore, we prepared a series of referenced catalysts like P–Co–NG, NG, Co_3_O_4_/GO composites, and Co_3_O_4_ for comparison. CV measurements reveal that P–Co–NG and NG show weak response toward UA oxidation without well-defined redox peaks, Co_3_O_4_/GO and Co_3_O_4_ can barely catalyze UA reaction (Fig. S8). The peak potential of UA oxidation can be used to judge the intrinsic electrocatalytic activity of the UA sensing electrode. The more negative anodic peak potential, the higher electrocatalytic activity. Figures 3a and S8 show that the peak potentials of UA oxidation for A–Co–NG, Co_3_O_4_, and P–Co–NG are 0.16, 0.52, and 0.54 V, respectively, of which the oxidation potential of A–Co–NG sensing anode is more negative than that of Co_3_O_4_ and P–Co–NG by 0.36 and 0.38 V, respectively, clearly indicating that A–Co–NG electrode has much higher electrocatalytic activity than the latter two. Amperometric *I* − *t* response is applied to systematically study the oxidase-like activities of various catalysts as shown in Fig. 3b. The A–Co–NG nanozyme exhibits the highest oxidase-like activity with a sensitivity of 301.6 μA mM^−1^ cm^−2^. Besides, the experimental order of oxidase-like activity is A–Co–NG > P–Co–NG > Co_3_O_4_/GO > Co_3_O_4_, indicating the intrinsic superiority of single-atom nanozymes (Fig. 3c).

Effect of pH on performance of the A–Co–NG toward UA oxidation was investigated. Result in Fig. S9 shows that the response of A–Co–NG sensor increases with increase in the pH from 10 to 13, reaching the highest response at pH 13. When the pH further increased to 14, the response decreases significantly. Thus, NaOH solution with pH of 13 was selected as the optimized condition for further investigation.

We further measured the cyclic voltammograms of A–Co–NG toward 500 μM UA in 0.1 M NaOH (pH = 13) at various scan rates form 0.03–0.4 V s^−1^. The anodic peak currents were found to be a linear function of the square root of scan rate with a linear regression equation as *I*_pa_ = 1.14 × 10^−4^ ν^1/2^–3.14 × 10^−6^ as shown in Fig. 3d. According to the relation of anodic peak current (*I*_P_a) versus square root of scan rate (*ν*^1/2^), an electron transfer number of 2 was obtained in terms of the equation [39] as follows:

$$I_{P} a = 2.69 \times 10^{5} \times \, \left( {D_{0} } \right)\cdot C_{0} \cdot A \cdot \nu^{1/2} \cdot n^{3/2}$$

where *D*_0_ is the diffusion coefficient, which is 7.5 × 10^−6^ cm^2^ s^−1^ for 500 μM UA [40]; *C*_0_ is the concentration of UA; A stands for electroactive surface area of the electrode, of which the calculated value is 0.0998 cm^2^ using [Fe(CN)_6_]^3−^/[Fe(CN)_6_]^4−^(5 mM) as a probe (data not show); n is the electron transfer number.

**Fig. 4:**
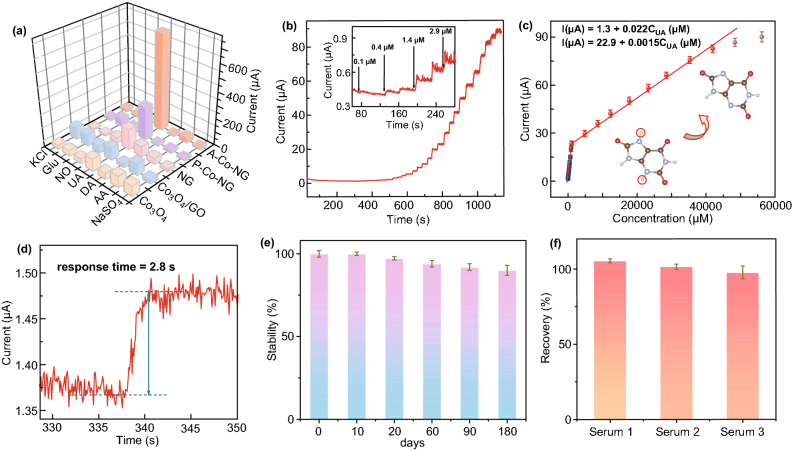
**a** Histogram of selectivity for UA detection of A–Co–NG, P–Co–NG, NG, Co_3_O_4_/GO and Co_3_O_4_; **b** Amperometric *I*−*t* curve of A–Co–NG upon continuous injection of different concentrations UA at an applied potential of 0.3 V versus SCE in 0.1 M NaOH (pH = 13); **c** Calibration plots of the A–Co–NG for UA determination with two linear ranges; **d** Amperometric *I−t* Response time of the A–Co–NG for UA determination; **e** Stability of A–Co–NG for UA detection with a long lifetime; **f** Recovery investigation of A–Co–NG performed by adding standard UA in human serum samples

Moreover, under oxidizing conditions, the presence of antioxidant species, such as AA, DA, NO, and so on can interfere with the UA detection in biological applications. The selectivity of the A–Co–NG and referenced catalysts toward UA oxidation was examined using amperometric method at 0.3 V versus SCE by analyzing various potential interfering species coexisting with UA, such as AA, DA, Glu, NO, K^+^, Na^+^, SO_4_^2−^, and Cl^−^. The current responses of these molecules, a key evaluate measurement for the specificity of proposed sensors, were summarized in Fig. 4a. Results show A–Co–NG (Fig. S10) performs the best selectivity and anti-interference ability with the presence of mixed or single AA, DA, Glu, NO, K^+^, Na^+^, SO_4_^2−^, and Cl^−^, which do not cause any noticeable interference to the UA response with the current signals relative standard deviation (RSD) less than 5%.

The amperometric *I*−*t* response of A–Co–NG upon successive addition of UA to a continuous stirred NaOH (0.1 M, pH = 13) was recorded. The influence of applied potential controlled from 0.1 to 0.4 V versus SCE on response of the A–Co–NG toward 33 μM UA was investigated (Fig. S11). The amperometric currents gradually increased along with the increasing of potential and exhibited a sharp increase at 0.3 V versus SCE. Considering the interference of many coexisted foreign species at too positive potential, 0.3 V versus SCE was chosen as the working potential to maintain a high sensitivity. As shown in Fig. 4b, the proposed sensor exhibits a rapid stepped increase response for the injection of UA. Figure 4c displays the calibration curve of the A–Co–NG for UA determination with two linear ranges from 0.4 to 1055 and 1055 to 41950 μM, with linear equations as *I* (μA) = 1.3 + 0.022 *C*_UA_ (μM) and *I* (μA) = 22.9 + 0.0015 *C*_UA_ (μM) at a correlation coefficient of 0.9981 (*n* = 24) and 0.9986 (*n* = 7), respectively. A low detection limit of 33.3 ± 0.024 nM is achieved, which is estimated from the expression of LOD = 3 S/K, where S is the standard deviation of the blank signals (*n*_*B*_ = 20), K is the analytical sensitivity that can be estimated from the slope of calibration curve at lower concentration ranges. The accomplished sensitivities of A–Co–NG nanozyme calculated from slopes of the calibration curves are 297.2 and 21.2 μA mM^−1^ cm^−2^, respectively. The calculated limit of quantitation (LOQ) of A–Co–NG for UA detection is 400 nM. Moreover, A–Co–NG can give a much wider linear range and a lower detection limit than the reported materials (Table 1). Besides, the as-prepared sensor achieves 95% of the steady-state current within less than 3 s (Fig. 4d). The short response time may be attribute to the fast adsorption of UA by the single Co atom catalyst. Furthermore, A–Co–NG nanozyme exhibits good stability by retaining above 90.5% activity after store for 180 days (Fig. 4e), indicating a good shelf-lifetime. By assaying 400 µM UA with five prepared sensors in same experiment conditions, the calculated RSD was 1.38%, indicating a satisfactory reproducibility and repeatability of this sensor. The reversibility of the UA sensor was also investigated, which can retain the response with a low RSD of 0.17% after testing for 10 times, indicating a good reversibility.

**Table 1:** Comparison of the performance of the previous studies and this work

Materials	Linear range (μM)	LOD (nM)	References
PB/N-doped CNTs	1–1000	260	[13]
Polyacrylamide-coated CNT	100–1000	–	[16]
GOx-CHIT/Co_3_O_4_ hollow nanopolyhedrons	0.3–3	100	[41]
Graphitic C_3_N_4_	10–100	8900	[42]
E-RGO	0.5–60	500	[43]
UOx/carbon ink printed electrodes	200–1000	–	[44]
SiO_2_/AuNP/PANI	5–1100	2000	[45]
Fe-Meso-PANI	10–300	5300	[46]
PANI-ABSA(p-aminobenzene sulfonic acid)	50–250	12,000	[47]
Polytetraphenylporphyrin/PPy/GO	5–200	1150	[48]
MoS_2_/poly(3,4-ethylenedioxythiophene) nanocomposite	2–25	950	[49]
AuNPs@ N-doped porous carbonaceous materials	1–150	100	[50]
MWCNT/PSVM/Au	0.05–1000	50	[51]
PEDOT/GCE	6–100	7000	[52]
rGO-ZnO	1–70	330	[53]
CeO_2-x_/C/rGO	49.8–1050	2000	[54]
AuNPs/MoS_2_-NSs	5–260	500	[55]
Polydopamine/Polypyrrole	0.5–40	100	[56]
A–Co–NG nanozyme	0.4–1055 and 1055–41,950	33.3 ± 0.024	This work

*UO*_*x*_ uricase oxidase, *GO*_*x*_ glucose oxidase, *PEDOT* poly(3,4-ethylenedioxythiophene

Serum examination is a convenient, safe, and inexpensive way to diagnose some diseases. To explore the potential applications of the single-atom nanozyme sensor toward UA, standard addition method was applied for several serum samples examination. The results are summarized in Table S2. As shown in Figs. 4f and S12, the recoveries ranged between 97.7 and 105.5%, indicating its practical application for analyzing UA in real biomedical samples. Besides, the results in real serum samples detected by this A–Co–NG sensor were compared with the standard assay conducted by a fully automatic biochemical analyzer (HITACHI LABOSPECT 008). The calculated accuracy of this sensor was 98.5% (RSD = 5.3%).

### Theoretical Study on Enzyme-like Activity of A–Co–NG

**Fig. 5:**
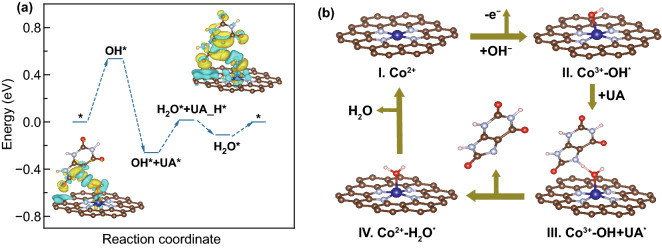
**a** Gibbs free energy profile for the UA oxidation pathways on A–Co–NG nanozyme. Inset of **a** are the CDD isosurfaces of Co^3+^–OH + ^*^UA before and after N–H bond dissociation (Co^2+^–H_2_O + ^*^UA_H) under oxidation potential (UA_H represent the structure of UA molecular after dehydrogenation of one H atom). For the contour plots, the charge accumulation regions are rendered in yellow, while the charge depleted regions are shown in cyan. The contour value of the CDD is ± 0.02 e Å^−3^. **b** Proposed mechanism of the oxidation process of UA on A–Co–NG nanozyme

To understand the interaction of A–Co–NG with UA analyte, the adsorption energies of UA on Co atom in A–Co–NG with vertically and parallel adsorption manner were calculated by DFT method (Fig. S13). The DFT results display long interaction distance of 2.31 and 2.38 Å for vertically and parallel adsorption configurations of UA on Co atom in A–Co–NG, respectively, indicating that interaction between UA and Co atom of A–Co–NG is weak. According to earlier study, A–Co–NG in aqueous solution were usually terminated by hydroxyl anion (OH^−^) group accompanying the Co^2+^ oxidized to Co^3+^ [25]. In our experiment, based on relation between oxidation peak of Co atom and catalytic active of A–Co–NG (Fig. 3a), we also find the catalytic activity originates from Co^3+^ rather than Co^2+^. The CV curve of A–Co–NG nanozyme (Fig. 3a) showed the center Co atom oxidizes from Co^2+^ to Co^3+^ by a OH^−^ at positive bias ~ 0.3 V versus AgCl, resulting in the formation of Co^3+^–OH structure, which is the same as the first step of oxygen evolution reaction (OER) in alkaline media. In process of OER on single Co atom catalyst, the step of second electron transfer (from ^*^OH to ^*^O) with a larger energy barrier (1.23 + 0.52 eV) is a rate limiting step [57], whereas formation of Co^3+^–OH–UA* state is energetic favorable with free energy of −0.796 eV, as shown in Fig. 5a. After formation of Co^3+^–OH–UA* state, the charger redistribution in the system happens under the driving force of oxidation potential. The insets of Fig. 5a show the charge density differences (CDD) isosurfaces of Co^3+^–OH + ^*^UA and Co^2+^–H_2_O + *UA_H states, respectively. It is obvious electron transfers from UA to Co^3+^–OH, which results in N–H bond dissociation, and a reduction of center Co atom from +3 to +2. The calculated energy barrier is 0.3 eV for Co^3+^–OH + ^*^UA state transferring to Co^2+^–H_2_O + *UA_H (Fig. 5a), and the desorption of *UA_H from Co^2+^–H_2_O is energetic favorable. Finally, followed by a H_2_O desorption with free energy of 0.14 eV, the A–Co–NG nanozyme returns to its initial state. The proposed mechanism of the oxidation process of UA on A–Co–NG nanozyme is shown in Fig. 5b. Overall, A–Co–NG nanozyme possesses excellent catalytic activity for UA oxidation, and the generation of Co^3+^–OH is the potential limiting step for UA oxidation.

The proposed catalytic mechanism was further confirmed by the comparison of the catalytic activity of A–Co–NG nanozyme at different states, in which the atomic Co center in +2 and +3 valence state, respectively. As shown in Fig. 3a, CV curves of the A–Co–NG in 0.1 M NaOH demonstrate the single Co atom mainly presents as low-valent Co (II) anchored on N-doped graphene, which was subsequently oxidized to Co (III) with the driving force over 0.3 V. The amperometric *I*-*t* curves were recorded with selected bias voltages at −0.05 and 0.4 V, corresponding two states of catalytic Co atoms, Co (II) and Co (III), respectively. As shown in Fig. S14a, the UA oxidation current for biased at 0.4 V is about 3 times of that of at -0.05 V, indicating higher UA oxidation catalytic activity of the *OH^−^ assistant reaction pathway. Moreover, although the redox behavior is not obvious, the potential-dependent UA detection performances of other cobalt-based samples (P–Co–NG) are similar with that of A–Co–NG nanozyme (Fig. S14b), which indicate that the catalytic mechanism of UA oxidation on the A–Co–NG is a general mechanism for UA oxidation.

## Conclusion

In summary, we report a single-atom catalyst A–Co–NG offering atomically dispersed Co–N center sites for building an electrochemical biomimetic sensor to highly sensitively and selectively detect UA. The A–Co–NG sensor also demonstrates its application in accurate serum examination toward UA, holding a great promise to its practical application in analysis of UA in real samples. This work provides a promising material with high active site density to realize UA detection with wide detection range and low detection limit, and the mechanism finding could be used to design and fabricate other kinds of SACs with enzyme-like activities for a wide range of biomimetic applications.

## Electronic Supplementary Material

Below is the link to the electronic supplementary material.

Supplementary material 1 (PDF 1165 kb)
