# Supplementary material for: Single-Atom Cobalt-Based Electrochemical Biomimetic Uric Acid Sensor with Wide Linear Range and Ultralow Detection Limit
Source: Nanomicro Lett. 2020 Oct 27;13:7. doi: 10.1007/s40820-020-00536-9 (PMC8187548; doi:10.1007/s40820-020-00536-9)
Supplement: Supplementary file 1 — Supplementary material 1 (PDF 1165 kb) [file 40820_2020_536_MOESM1_ESM.pdf]

Supporting Information for

Single-Atom Cobalt-Based Electrochemical Biomimetic Uric Acid Sensor with Wide Linear Range and Ultralow Detection Limit

Fang Xin Hu[1], Tao Hu[1], Shihong Chen[4], Dongping Wang[5], Qianghai Rao[1], Yuhang Liu[1], Fangyin Dai[6], Chunxian Guo[1, *], Hong Bin Yang[1, *], Chang Ming Li[1, 2, 3, *]

[1]Institute of Materials Science & Devices, Suzhou University of Science and Technology, Suzhou 215009, People's Republic of China

[2]Institute for Advanced Cross-field Science & College of Life Science, Qingdao University, Qingdao 200671, People's Republic of China

[3]Institute for Clean Energy & Advanced Materials, School of Materials and Energy, Southwest University, Chongqing 400715, People's Republic of China

[4]School of Chemistry and Chemical Engineering, Southwest University, Chongqing 400715, People's Republic of China

[5]Suzhou Institute of Biomedical Engineering and Technology, Chinese Academy of Sciences, Suzhou 215163, People's Republic of China

[6]State Key Laboratory of Silkworm Genome Biology, College of Biotechnology, Southwest University, Chongqing 400715, People's Republic of China

*Correspondence authors. E-mail: cxguo@usts.edu.cn (CX Guo); yanghb@usts.edu.cn (HB Yang); ecmli@swu.edu.cn (CM Li).

Supplementary Figures and Tables

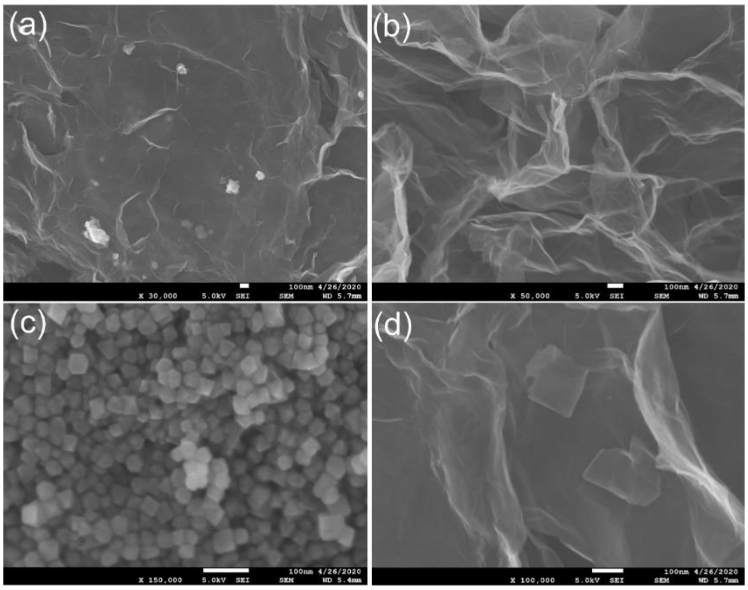

Fig. S1 SEM image of (**a**) P-Co-NG; (**b**) NG; (**c**) Co_3O_4; (**d**) Co_3O_4/GO

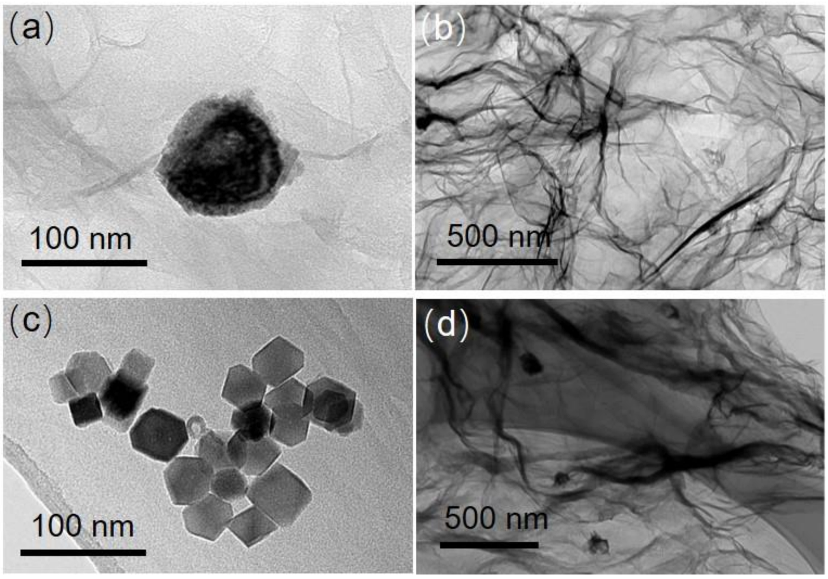

Fig. S2 TEM image of (**a**) P-Co-NG; (**b**) NG; (**c**) Co_3O_4; (**d**) Co_3O_4/GO

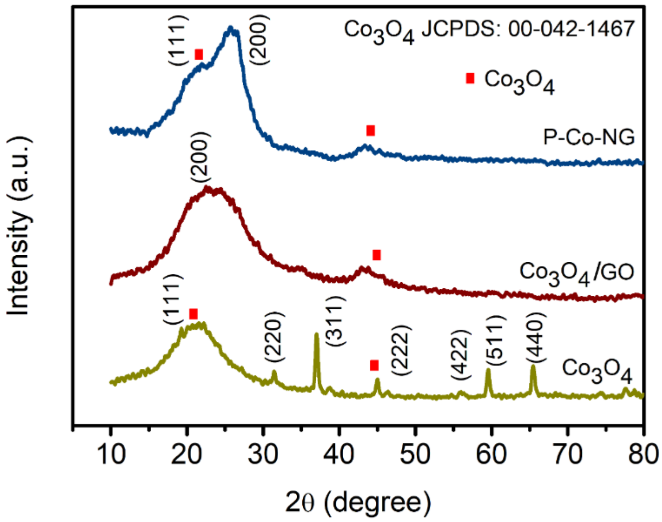

Fig. S3 XRD patterns of materials

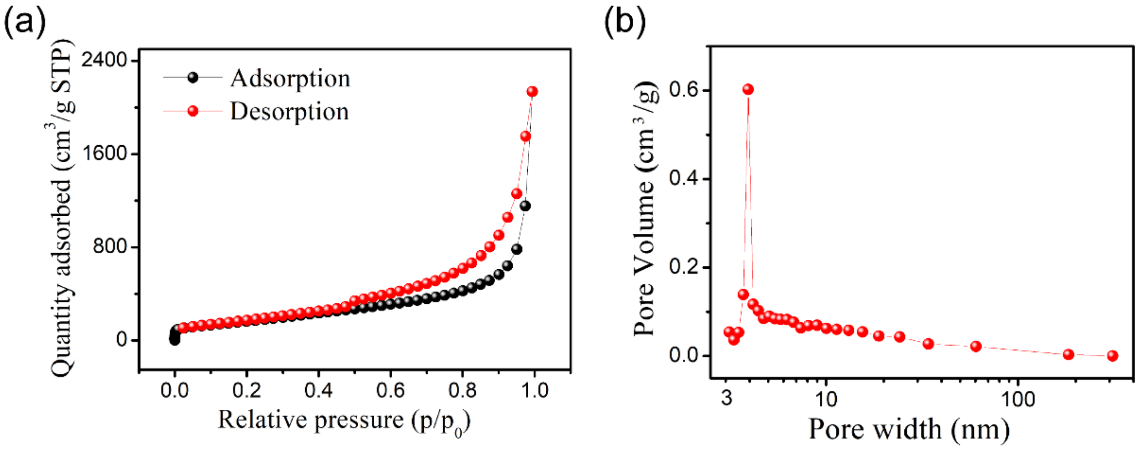

Fig. S4 BET analysis of A-CO-NG

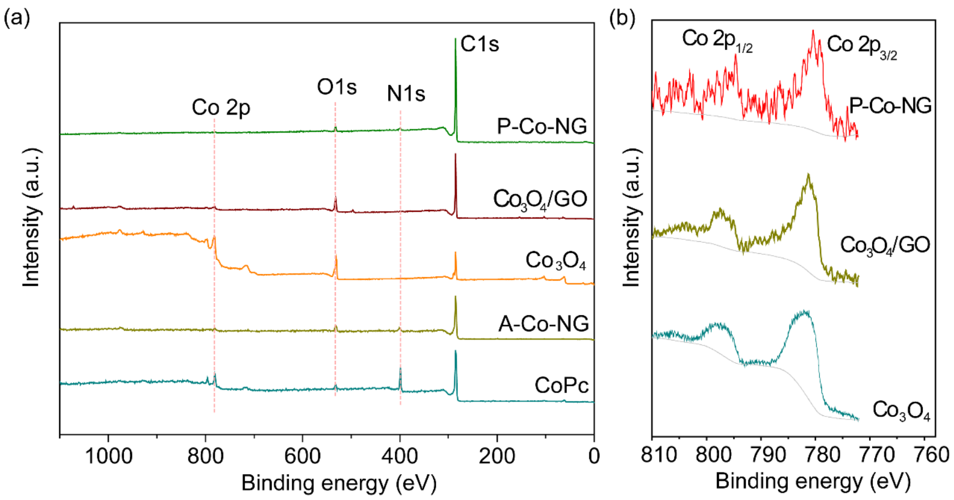

Fig. S5 XPS analysis of different samples: (**a**) full spectra; (**b**) Co 2p spectra

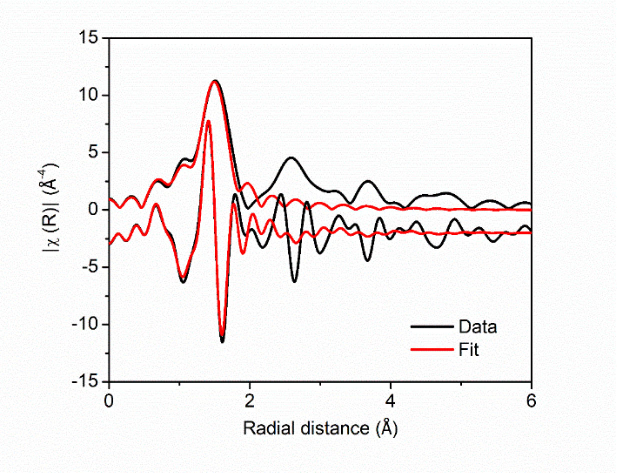

Fig. S6 First-shell fitting of the Fourier transformation of the EXAFS spectrum of Co-Pc (the EXAFS spectrum was fitted using the FEFF 8.2 code)

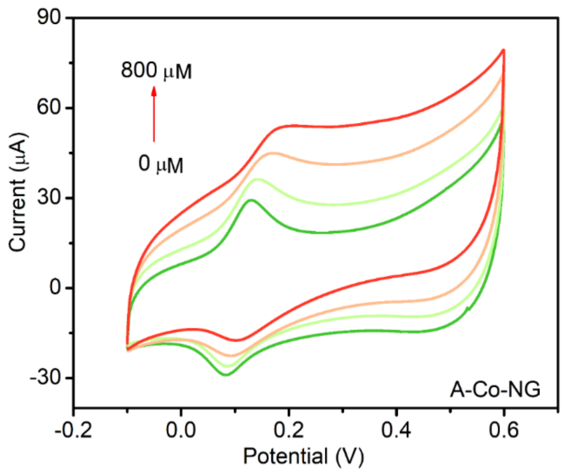

Fig. S7 CV curves of the A-Co-NG nanozyme recorded in a 0.1 M NaOH solution with increasing UA concentration

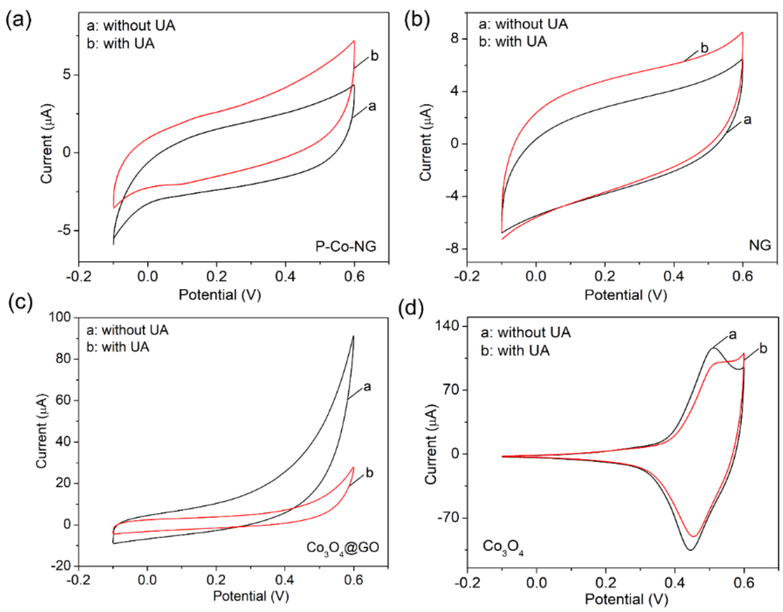

Fig. S8 CV curves of various catalysts towards **a**) without and **b**) with 400 μM UA recorded in a 0.1 M NaOH solution

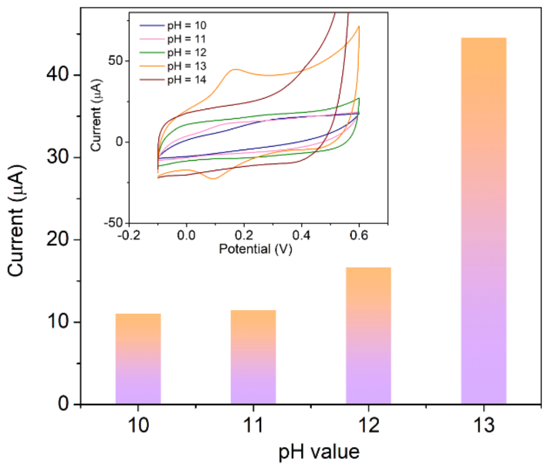

Fig. S9 The influence of pH value of test solution controlled from 10 to 14 on the electrochemical response of the A-Co-NG towards 400 μM UA

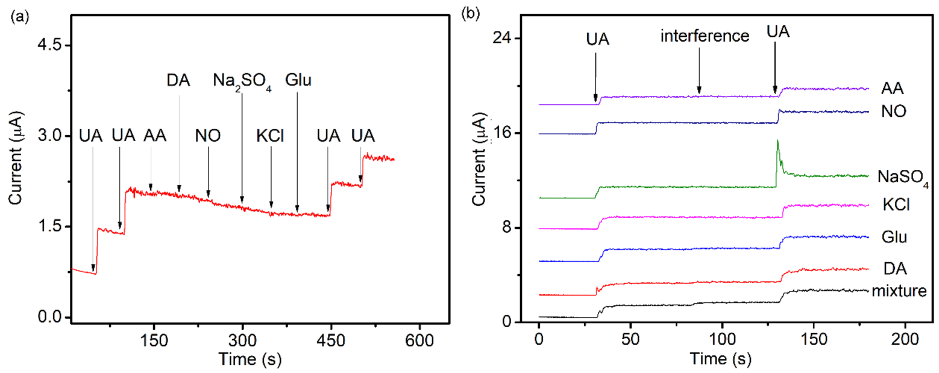

Fig. S10 (**a**) Selectivity for UA detection of A-Co-NG nanozyme. (**b**) Selectivity for UA detection of A-Co-NG with mixed or single interfering species

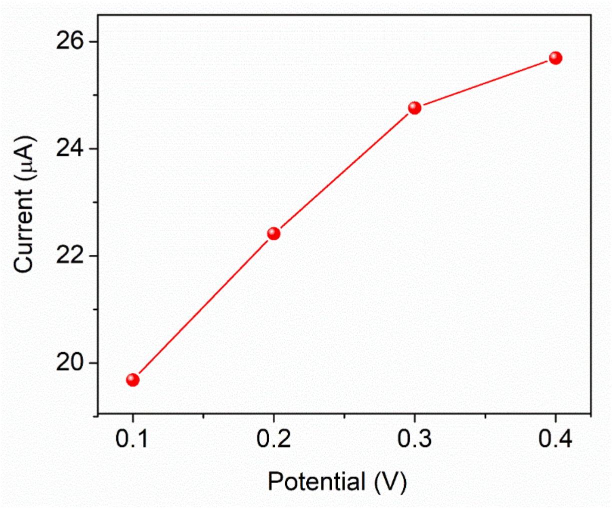

Fig. S11 The influence of applied potential controlled from 0.1 to 0.4 V vs. SCE on current response of the A-Co-NG towards 33 μM UA

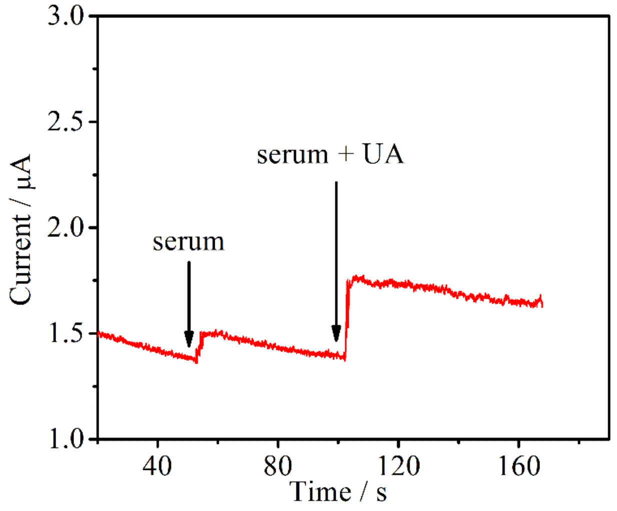

Fig. S12 Amperometric *I-t* response of A-Co-NG for serum sample detection

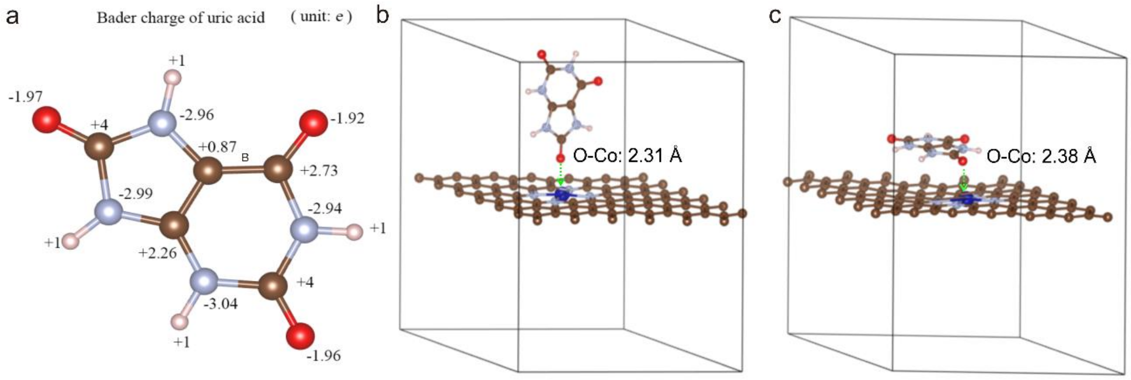

Fig. S13 (**a**) Bader charge of UA; Adsorption of UA molecule on A-Co-NG with optimized configuration of (**b**) vertical adsorption and (**c**) parallel adsorption

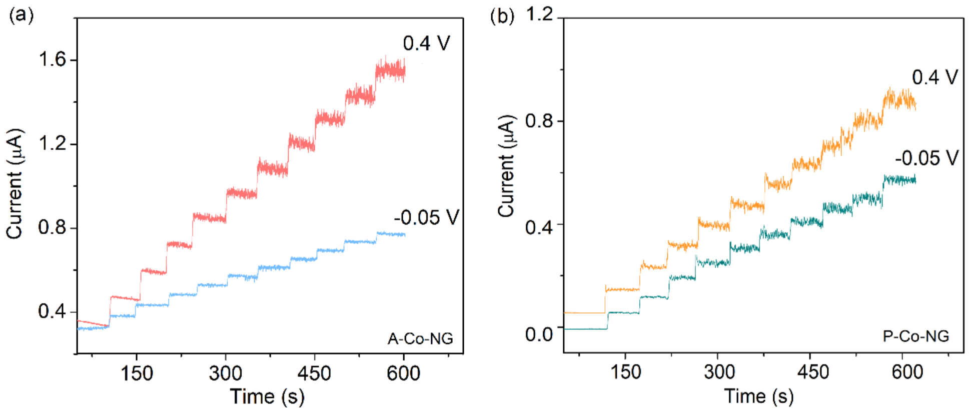

Fig. S14 Amperometric I-t response of (**a**) A-Co-NG and (**b**) P-Co-NG upon continuous injection of 5 μM UA at an applied potential of 0.4 and -0.05 V vs SCE in 0.1 M NaOH

Table S1 Best fitting EXAFS data for different Co-based catalysts

**Sample**	**Shell**	**N**	**R (Å) (EXAFS)**	**ΔE (eV)**	**Debye-Waller factor Δσ2 (Å^2)**	**R-factor**
Co-N-G	Co-N	3.38±0.67	1.87±0.02	-3.6 ±2.4	0.005±0.002	0.0055
CoPc	Co-N	3.58±0.52	1.9±0.03	-3.6 ±3.0	0.002±0.0034	0.0085

N: coordination number.

Table S2 Results for the determination of UA in real samples

**Sample**	**Detected (μM)**	**Added (μM)**	**Found (μM)**	**Recovery (%)**
Serum 1	1.90	9.80	12.24	105.5
Serum 2	1.00	9.80	10.58	97.7
Serum 3	2.29	9.80	12.25	101.6
